# Microinjection molded microwell array-based portable digital PCR system for the detection of infectious respiratory viruses

**DOI:** 10.1186/s40580-025-00482-5

**Published:** 2025-03-21

**Authors:** Ji Wook Choi, Daekyeong Jung, Yoo Min Park, Nam Ho Bae, Seok Jae Lee, Donggee Rho, Bong Geun Chung, Kyoung G. Lee

**Affiliations:** 1 Department of Mechanical Engineering, Sogang University, 35 Baekbeom-ro, Mapo-gu, Seoul, 04107 Republic of Korea; 2 Institute of Integrated Biotechnology, Sogang University, 35 Baekbeom-ro, Mapo-gu, Seoul, 04107 Republic of Korea; 3 Center for Nano-Bio Development, National NanoFab Center (NNFC), 291 Daehak-ro, Yuseong-gu, Daejeon, 34141 Republic of Korea; 4 Institute of Smart Biosensor, Sogang University, 35 Baekbeom-ro, Mapo-gu, Seoul, 04107 Republic of Korea

**Keywords:** Microfluidic chip, Microarray, Digital PCR, Injection mold, COVID-19

## Abstract

In molecular diagnostics, the digital polymerase chain reaction (dPCR) has been considered a promising point-of-care testing (POCT) method for the rapid and accurate analysis of respiratory infections. To improve its practical applicability, it is necessary to develop a mass-producible and reproducible dPCR system for nucleic acid partitioning; additionally, the system must provide a customized portable analysis. In this study, we report an advanced mass-production method for the fabrication of microwell array-based dPCR chips suitable for nucleic acid partitioning and a compact fluorescence signal analysis dPCR system. Based on metal mold fabrication, different microwell sizes with diameters in the 100–200 μm range and pitches in the 200–400 μm range are designed and successfully fabricated using photolithography, metal electroplating, and injection molding techniques. Additionally, a battery-operated dPCR system utilizing digitalized fluorescence signal analysis is developed for on-site detection. To verify the chip and system applicability, the infectious human coronavirus is analyzed using different nucleic acid concentrations. By evaluating the performance of the dPCR chips and system, accurate and quantitative virus analysis results are obtained, verifying the portability, easy use, and reproducibility of the chips and system. Furthermore, the detection results obtained using the fabricated chips and the developed system are similar to the results obtained using commercially available systems, verifying that the proposed dPCR chips and system exhibit sensitivity, accuracy, reliability, and reproducibility in the quantitative molecular analysis of infectious diseases.

**Graphical Abstract:**

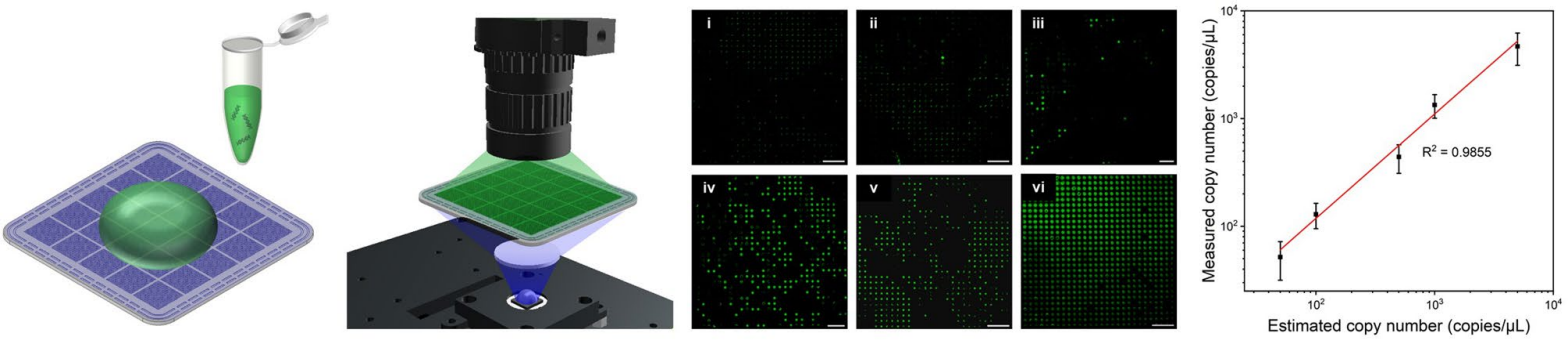

## Introduction

The digital polymerase chain reaction (dPCR) has attracted interest as an advanced PCR-test format and applied in various fields such as biological research and clinical diagnostics [1–4]. Due to its high sensitivity, precision, absolute quantification, and accuracy, dPCR has become an important and widely used nucleic acid detection technique [5–8]. To this day, various dPCR platforms, such as the microwell-based dPCR and droplet-based dPCR platforms, have been successfully developed and commercialized [9–11]. Typically, a dPCR platform includes a dPCR chip and a system. dPCR chips are required to perform numerous functions including compartmentalization, thermal amplification, and transparency for optical analysis [12]. So far, silicon-based microarray chips have been precisely fabricated and commercialized for use in microwell-based dPCR platforms using photolithography [13–15]. Despite the significant efforts made, previously reported techniques require clean room facilities with expensive instruments; additionally, they exhibit difficulties in massive fabrication. Additional requirements for a practically applicable dPCR system include compactness, accurate fluorescence signal analysis, and portability [16–18]. Therefore, cost-effective methods for fabricating highly uniform, reproducible, and reliable plastic-based microwell chips are highly required to expand the applicability of dPCR systems.

Traditional fabrication methods of microwell array-based chips, such as computer numerical control micromilling, soft lithography, and three-dimensional (3D) printing, have been widely applied to overcome the above problems because they provide rapid accessibility, inexpensive fabrication for low quantities, and suitability in the early-stage chip development phase [19–24]. However, these methods require multiple manual preparation steps, resulting in high fabrication costs and chip-to-chip variability. Additionally, many prototyping techniques require specific materials (e.g., polydimethylsiloxane for soft lithography and ultraviolet (UV)-curable resin for 3D printing). Furthermore, they can lead to tool marks that can render a chip incompatible with dPCR research applications due to low transparency, gas permeability, gas bubble formation, and evaporation of reagents [25–28]. Recently, injection molding has become popular in the fabrication of plastic-based chips for molecular diagnostics including dPCR [29, 30]. This technique provides high-throughput chip manufacturing, relatively low fabrication cost, high reproducibility, and biocompatibility [31–33]. Furthermore, it enables the production of highly transparent and low autofluorescence polymers (e.g., cyclic olefin copolymer, polycarbonate, polymethyl methacrylate) [34–36]. Although the injection molding technique has numerous advantages, there is a need for advanced and precise microstructured array-based metal molds.

Further issues need to be addressed in the design of current dPCR systems, particularly when applied to point-of-care testing (POCT). These systems often involve sophisticated and bulky instruments, which constrain their portability and ease of use [37]. In addition, the necessity for precise thermal cycling and high-resolution fluorescence detection increases the complexity and cost of these systems, thus limiting their accessibility. To overcome these limitations, portable dPCR systems have recently been developed by incorporating miniature components including heating elements and image sensors [38–41]. Nevertheless, the integration of these components into a compact and user-friendly system remains a significant challenge, particularly in maintaining the required precision and stability during operation.

Considering all the above requirements, we developed mass-producible microwell array-based chips and a fluorescence analysis dPCR system using microelectromechanical-based metal molds and injection molding methods. To fabricate the chips, we used a polished nickel (Ni) plate to construct highly arrayed micropatterns based on photolithography and electroplating in the manufacturing of metal molds. Additionally, polycarbonate (PC) was used in the fabrication of dPCR chips because of its chemical and mechanical stability and high transparency. The fluorescence analysis system was designed to integrate essential functions, including thermal cycling, fluorescence imaging, and digital analysis. The system, which was constructed using a complementary metal-oxide semiconductor (CMOS) sensor, is compact, making it suitable for easy transportation and deployment in various environments. In addition, it operates on minimal power, allowing for battery-powered operation, making it suitable for field applications. To verify the chip and system performance, an infectious respiratory virus of the human coronavirus 229E was used as a realistic sample. The highly microarrayed chips and the system demonstrated excellent and precise analysis performance, providing cost-effectiveness, transparency, sensitivity, reliability, and reproducibility.

## Materials and methods

### dPCR system fabrication

The proposed dPCR system was developed in our laboratory by making minor modifications to a previously developed system [42]. In our system, the thermal cycling module included a blue LED (LXML-PR02-1000, Lumileds, USA), a gold nanopillar array (Au NPA), and a cooling fan (Pi-FAN, ICBanq, Korea). The imaging module included a CMOS sensor (HQ camera, Adafruit, USA) with a focal lens (IMX477, Sony, Japan). A multicolor LED (LZC-03MD07, Osram, Germany) was employed as a light source. Excitation (ET490/20x, Chroma, USA) and emission (ET520/20m, Chroma, USA) bandpass filters were used for fluorescence imaging. The system was powered by lithium-ion batteries, and the entire process was controlled by a single-board computer (Raspberry Pi 4B, Adafruit, USA). The exterior of the system was designed using 3D computer-aided design software (Inventor, Autodesk, USA) and fabricated using a 3D printer (DP200, Sindoh, Korea).

### Microwell array-based chip fabrication

**Fig. 1:**
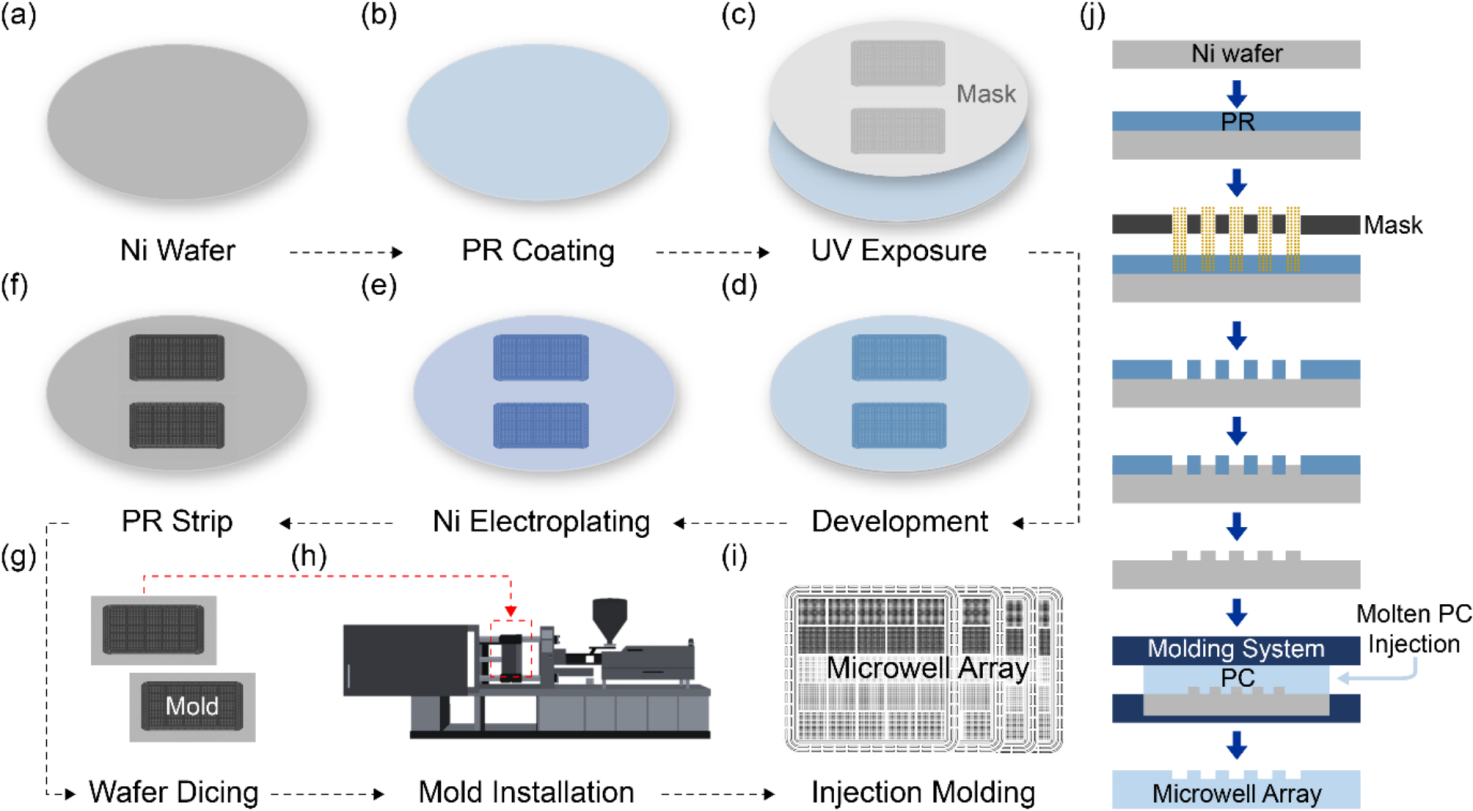
Schematic of the microwell array-based chip fabrication process. (**a**) 6-inch Ni wafer. (**b**) Photoresist coating applied on a cleaned Ni wafer. (**c**) UV light exposure using a photomask. (**d**) Photoresist development. (**e**) Ni-layer deposition using electroplating. (**f**) Photoresist removing. (**g**) Ni wafer dicing for mold fabrication. (**h**) Ni-mold installation in the plastic injection molding machine. (**i**) Microwell array-based chip fabrication. (**j**) Cross-sectional views of the fabrication process

Plastic injection molding employing a metal mold was used in the fabrication of microwell array-based chips. The mold was fabricated using photolithography and electroplating, as shown in Fig. 1. A 6-inch, 1-mm thick Ni wafer was prepared and cleaned to obtain a seed metal mold layer (Fig. 1a). Positive photoresist (THB-151 N) was spin-coated at 1,200 rpm for 40 s and baked at 120 °C for 10 min on a hot plate to fabricate microsized patterns on the Ni wafer (Fig. 1b). The photoresist-coated wafer was then placed under a photomask installed in a mask aligner and exposed to 890 mJ/cm^2^ UV light (Fig. 1c). The same process of photoresist coating to UV exposure was repeated to obtain a photoresist pattern height of more than 50 μm. After a second UV exposure, the wafer was immersed into a developer (DVL-200) for 8 min and cleaned using distilled water to create photoresist hole patterns with diameters of 100, 150, and 200 μm (Fig. 1d). The wafer was immersed into an electroplating solution at 55 °C for 225 min to deposit a ~ 60-µm thickness Ni-layer on the patterned region, where the Ni seed layer was exposed (Fig. 1e). The residual photoresist patterns were removed from the wafer surface using acetone (Fig. 1f), and the Ni wafer was diced into two metal mold pieces (Fig. 1g). The micropillar-patterned metal mold was then installed at the center of a mold in a plastic injection molding machine (Fig. 1h). PC was selected as the plastic material instead of thermoplastic polyurethane because of its better surface smoothness, clarity, and repeatability. The PC melt temperature was between 260 °C and 340 °C; the molten PC was injected into the molding machine by applying a pressure in the 70–140 MPa range, where the mold temperature was set to ~ 120 °C considering the glass transition temperature. After performing the plastic injection, a pressure of ~ 150 MPa was applied to the molds for 20 s, and the microwell array-based chips were released from the molding system, finalizing the fabrication process (Fig. 1i). Figure 1j shows cross-sectional views of the entire chip fabrication process.

### Microwell array-based chip modification

The developed microwell array-based chips were treated using oxygen plasma (CUTE, Femto Scientific, Korea) and subsequently coated with hydrophilic agents to improve sample retention and reduce nonspecific interactions. Polyvinyl alcohol (PVA, Sigma Aldrich, USA), S100 (Joninn, Denmark), and P100 (Joninn, Denmark) were placed on the chip surface to investigate the hydrophilic coating effect. The chips were incubated in an oven at 85 °C for 2 h. A fluorescein isothiocyanate (FITC, Sigma Aldrich, USA) solution was used to determine the sampling efficiency and evaporation rate of the chips. The sampling efficiency was calculated by dividing the number of filled microwells by the total number of microwells.

### dPCR assay

The human coronavirus HCoV-229E (alphacoronavirus, KBPV-VR-9) was obtained from the Korea Bank for Pathogenic Viruses. Subsequently, reverse transcription was conducted using a one-step RocketScript™ Reverse Transcriptase (Bioneer, Korea) according to the manufacturer’s instruction to synthesize complementary DNA (cDNA). Reaction mixtures consisted of a 100 µL master mix (AccuPower^®^ PCR Master Mix, Bioneer, Korea) containing 2 µL of template DNA, 2 µL of primers (10 µM), and 2 µL of TaqMan probes (10 µM) were used for dPCR analysis. The detailed sequences of primers and probes are presented in Table 1. Highly concentrated samples were serially diluted using nuclease-free water (Sigma Aldrich, USA). The concentration of the prepared master mix was determined using a Nanodrop 2000 spectrophotometer (Thermo Scientific, USA), and the mix was dispensed onto the microwell array-based chips; the chips were subsequently sealed using a PCR sealing tape (Sigma Aldrich, USA). The PCR thermal cycling protocol involved 40 cycles of denaturation at 90 °C for 10 s and combined annealing and extension at 60 °C for 35 s. Fluorescence images were acquired using the developed dPCR system, and the fluorescence intensities were determined using a custom-written algorithm, previously developed in our laboratory [39]. The concentration of the target DNA was calculated using the Poisson distribution:

$$\:P\left(k,\lambda\:\right)\:=\:\frac{{\lambda\:}^{k}{e}^{-\lambda\:}}{k!}$$

$$\:{f}_{p}\:=\:P\left(k\:>\:0\right)\:=\:1-P\left(k\:=\:0\right)\:=\:1-\:{e}^{-\lambda\:}\:$$

$$\:\lambda\:\:=\:-\text{ln}\left(1-{f}_{p}\right)\:$$

$$\:C\:=\:\frac{\lambda\:}{{V}_{microwell}}$$

where *P(k*,* λ)* is the probability of finding *k* target-DNA molecules in a given partition, *λ* is the average number of DNA molecules per microwell and *f*_*p*_ is the ratio of positive wells to the total number of microwells. To validate the proposed system, a comparative real-time PCR was conducted using a commercially available system (CFX96, Bio-Rad, USA) under identical conditions to those used in dPCR assay.

**Table 1:** Sequences of primers and probes used in the dPCR assay

Material	Sequence (5’→ 3’)
Forward primer	tgtttgatagtcacattgtttccaaagagt
Reverse primer	ttgaattctagtgcactagggttaagaaga
Probe	ggcaacactgtggtcttgactttcac tact[FAM-dT] a[THF]a[BHQ1-dT]gtgactgtgcccaaa[C3-spacer]

## Results and discussion

### Μicrowell array-based dPCR system operation

**Scheme 1:**
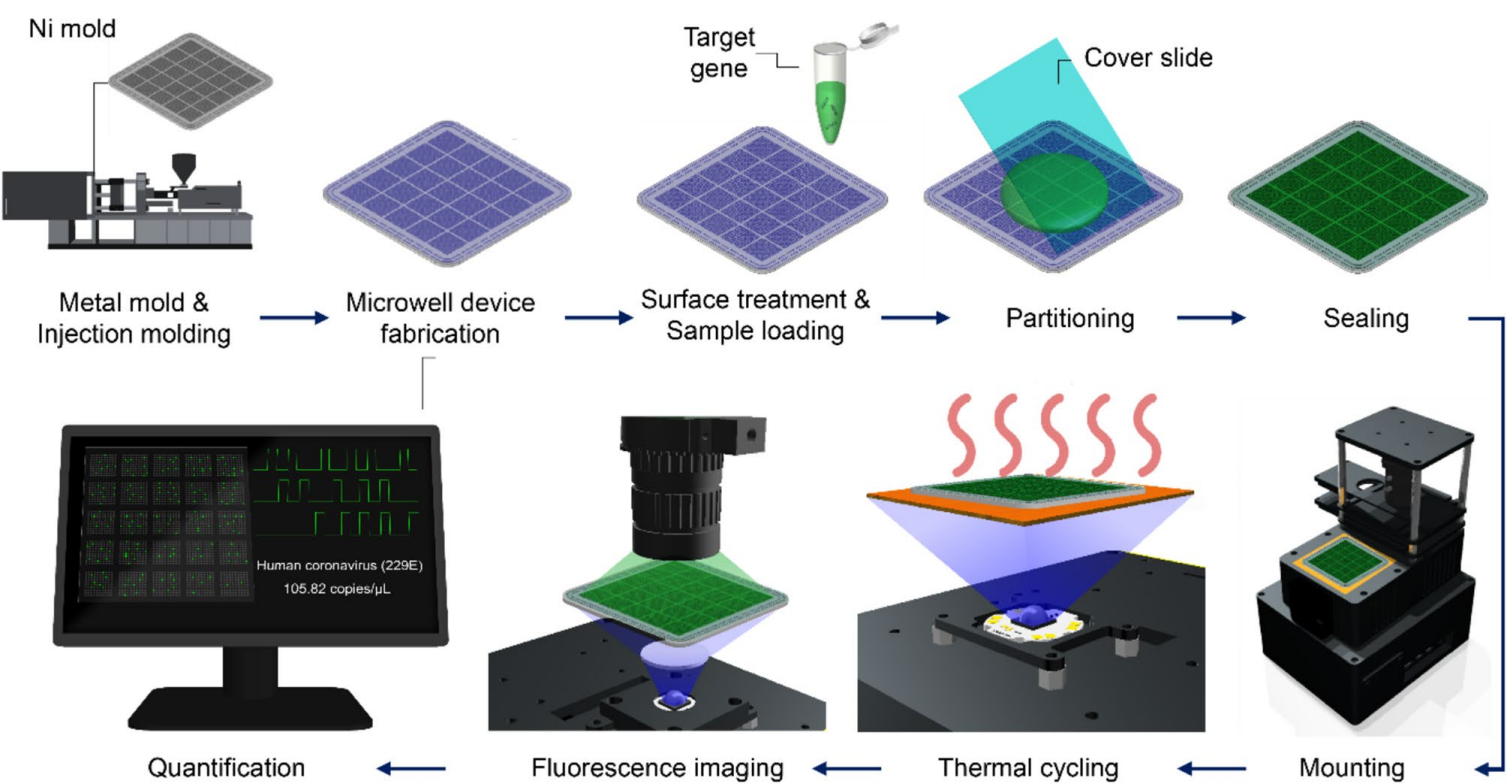
Overall microwell dPCR chip fabrication process and dPCR system operation

The overall microwell dPCR chip fabrication process and the operation of the developed microwell array-based dPCR system are schematically illustrated in Scheme 1. Initially, mirror images of microwell array patterns were fabricated; these patterns were then incorporated into a metal mold and carefully placed on an injection molding machine for massive and reproducible production. In this study, PC was used because of its high transparency, chemical reliability, and normally maintained rigidity up to 140 °C. The chip surface was then treated and coated using a hydrophilic agent for efficient sample loading on the microwells. The PCR reaction mixture, which contained the target gene, and the corresponding primers and probes were dispensed onto the microwells and partitioned into individual wells using a cover slide. After loading the sample, a transparent sealing tape was placed over the microwell array to prevent evaporation and cross-contamination between wells. The sealed array was then subjected to thermal cycling, involving repeated heating and cooling cycles, to enable the denaturation of DNA, annealing of primers, extension of the DNA strand, and amplification of the target DNA. Next, a fluorescence imaging system was used to capture images of the microwells to detect the presence of fluorescence. Finally, the images were analyzed to determine the target DNA concentration.

### Metal mold and microwell array-based chip fabrication

**Fig. 2:**
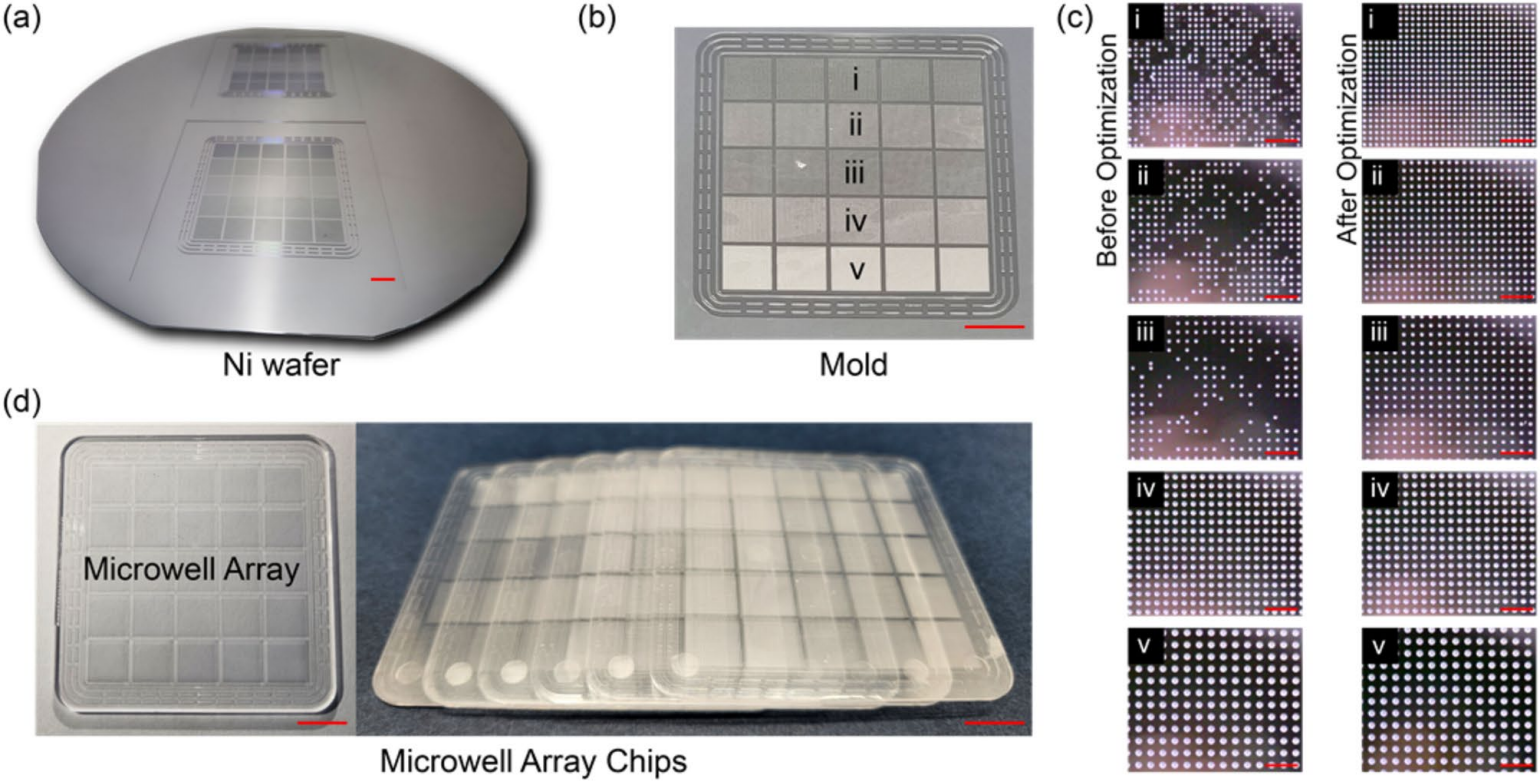
Fabrication of a Ni wafer consisting of two molds and microwell array chips. (**a**) Photograph of a Ni wafer (scale bar: 1 cm). (**b**) Photograph of a diced mold showing five different microwell array types (scale bar: 1 cm). (**c**) Microscope images of microwell arrays in a Ni-mold before and after photolithography optimization (scale bars: 1 mm). (**d**) Photograph of microwell array-based chips (scale bars: 1 cm)

**Fig. 3:**
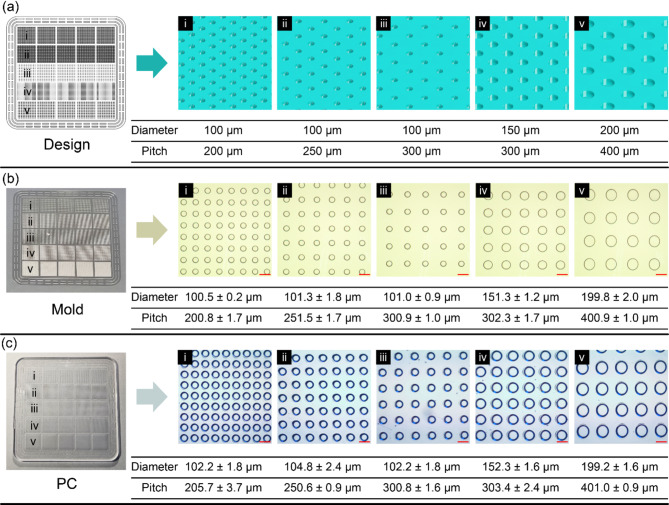
Inspection results of the fabricated microwell array-based chips including five microwell array types (types i–v). (**a**) Images of original design and corresponding dimensions. (**b**) Ni-mold microscope images and measured diameters and pitches. (**c**) Microscope images of a chip and measured diameters and pitches (scale bars: 200 μm)

A Ni wafer was fabricated using photolithography and electroplating (Fig. 2a). The wafer was diced into two metal molds (Fig. 2b), as depicted in Fig. 1a-g. The microwell array types in the metal mold are the following: (1) Type i (diameter: 100 μm; pitch: 200 μm). (2) Type ii (diameter: 100 μm; pitch: 250 μm); (3) Type iii (diameter; 100 μm; pitch: 300 μm); (4) Type iv (diameter: 150 μm; pitch: 300 μm); (5) Type v (diameter: 200 μm; pitch: 400 μm) (detailed images are shown in Fig. 3a). The diameters and pitches of the microwell array were determined by considering the resolution and reproducibility limitations of the metal mold used in plastic injection molding. Additionally, the selected dimensions were optimized to ensure appropriate sample volume loading and to minimize evaporation, which affect PCR assay performance, as further described in the below Sect. 3.5. In the fabrication process, the first version of the micropillar-patterned metal mold was fabricated and inspected using an optical microscope, as shown in the left-column images in Fig. 3c. The results showed that a 100-µm diameter (types i–iii) resulted in a ~ 50% defect rate compared to a 150-µm diameter (type iv) and a 200-µm diameter (type v). The mold defects, where the pillar patterns were missing, can be attributed to the not fully developed photoresist hole patterns and the Ni electroplating on top of the patterns. After optimizing the parameters, including baking time and exposure time to UV light in the photolithography, the defect rates in all five types of the microwell array were reduced to almost 0%, as shown in the right-column images in Fig. 3c. The evaluation results of the sampling efficiency and evaporation rate in the dPCR assays are described in the experimental section. Figure 3d shows the fabricated microwell array-based chips using the injection molding system with the optimized Ni-mold.

### Metal mold and microwell array-based chip inspection

The Ni molds and the PC-fabricated microwell array-based chips were further inspected using a contact surface profilometer and an optical microscope and analyzed by comparing the obtained data with the original design data, as shown in Fig. 3. The measured diameters of Ni-mold types i, ii, iii, iv, and v were measured to be 100.5 ± 0.2 μm, 101.3 ± 1.8 μm, 101.5 ± 0.9 μm, 151.3 ± 1.2 μm, and 199.8 ± 2.0 μm, respectively. The corresponding measured pitches were measured to be 200.8 ± 1.7 μm, 251.5 ± 1.7 μm, 300.9 ± 1.0 μm, 302.3 ± 1.7 μm, and 400.9 ± 1.0 μm, respectively. These measured Ni-mold diameters and pitches are uniform and close to the original design values. The mold microscope images show that the microsized pillar patterns are well-defined on clean surfaces with smooth edges, indicating that the mold fabrication process is accurate and reliable (Fig. 3b). The measured diameters of the microwell array-based chips of types i, ii, iii, iv, and v were measured to be 102.2 ± 1.8 μm, 104.8 ± 2.4 μm, 102.2 ± 1.8 μm, 152.3 ± 1.6 μm, and 199.2 ± 1.6 μm, respectively. The corresponding measured pitches were 205.7 ± 3.7 μm, 250.6 ± 0.9 μm, 300.8 ± 1.6 μm, 303.4 ± 2.4 μm, and 401.0 ± 0.9 μm, respectively. The measured diameters and pitches of the chips are in good agreement with the design specifications. The microscope images of the chips show that the patterns are uniform and well-ordered, and there are no significant surface defects or irregularities. These results clearly indicate that the PC-fabricated chips were successfully replicated by the injection molding system, and the fabrication process is well-controlled and repeatable, producing high-quality patterns with micron resolution. These chips exhibit high potential in future mass production.

### dPCR system development

**Fig. 4:**
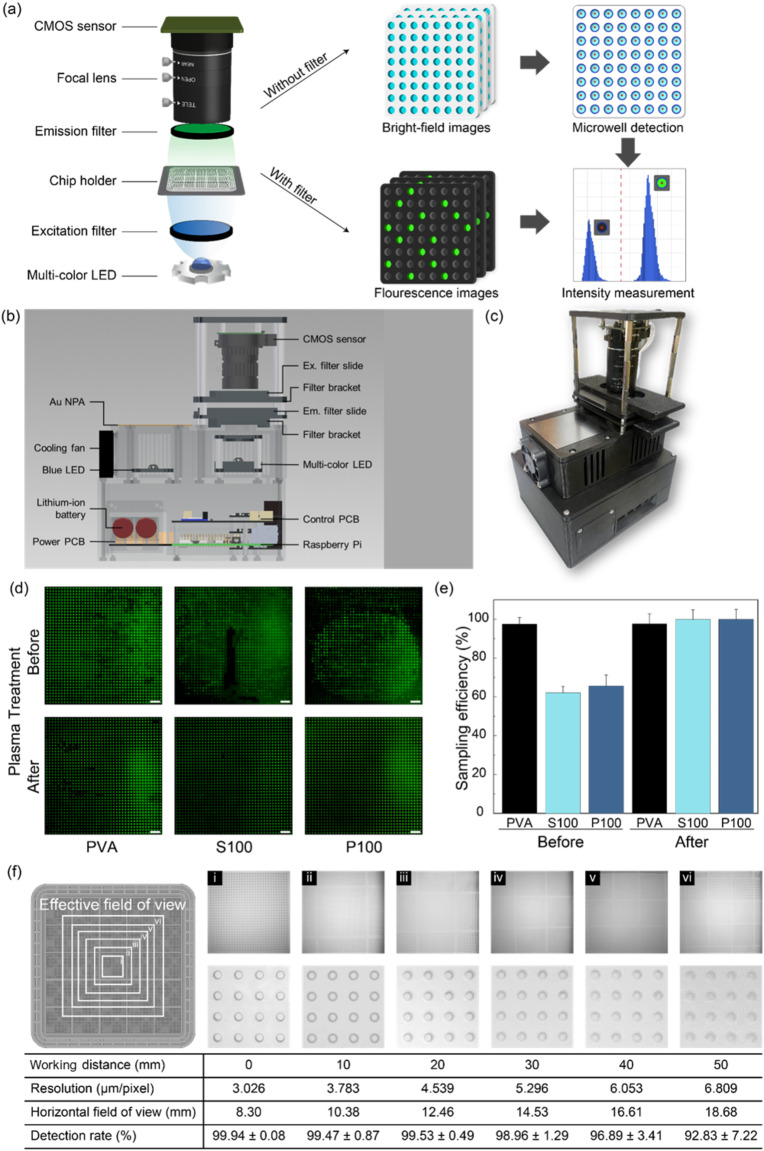
Comprehensive overview of the developed dPCR system. (**a**) Schematic of the image analysis module integrated into the dPCR system. (**b**) Vertical cross-sectional view of the entire dPCR assembly. (**c**) Photograph of the fabricated dPCR system and its optimization. (**d**) Fluorescence microscopy images of an FITC-loaded microwell array-based chip before and after surface modification (scale bars: 1 mm). (**e**) Sampling efficiency graph of a chip treated using three different types of hydrophilic agents. (**f**) Schematic representation of the effective field of view (FoV) at different working distances between the optical lens and the chip; bright-field images of the chip captured at different working distances: (i) 0 mm, (ii) 10 mm, (iii) 20 mm, (iv) 30 mm, (v) 40 mm, and (vi) 50 mm; table summarizing the resolution (µm/pixel), horizontal FoV (mm), and detection rate (%) at each working distance

To enable the practical applicability of the fabricated microwell chips, we developed a dPCR system with integrated thermal cycling and fluorescence imaging modules. The system was used in dPCR assays, as shown in Fig. 4. Figure 4a illustrates the fluorescence imaging process in the proposed dPCR system. In the thermal cycling module, the Au NPA was employed to achieve rapid and uniform heating. The Au NPA consisted of nanopillars with a diameter of 500 nm and a height of 500 nm, arranged with a center-to-center distance of 1.2 μm. When exposed by an LED, the array exhibits localized surface plasmon resonance (LSPR), which efficiently converts incident light into heat. This LED-driven plasmonic heating mechanism not only accelerates the thermal cycling process but also ensures precise temperature control across the microwell chip. After the thermal cycling process was completed, a fabricated microwell array-based chip was placed on the imaging module, and bright-field images were acquired to detect the location of microwell arrays. Subsequently, fluorescence images were obtained using fluorescence bandpass filters, and their corresponding intensities were measured for each microwell. The intensities were plotted on a histogram, and the threshold was automatically determined using a custom algorithm. Finally, the initial concentration of the target DNA was calculated using Poisson distribution statistics. Essential functions, such as thermal cycling and fluorescence imaging (Fig. 4b), were integrated into the dPCR system. A key innovation of this system is its compact size (12.8 × 15.7 × 20.6 cm), making it suitable for easy transportation and deployment in various environments (Fig. 4c). Additionally, the system operates on low power, requiring less than 20 W to operate efficiently. This combination of portability and low-power consumption enables the dPCR system to be battery-operated, making it suitable for field applications, where the use of conventional laboratory equipment is impractical.

### Optimization and evaluation of the dPCR system detection sensitivity

To optimize the performance of the dPCR system, we systematically modified essential functions including sample digitization, microwell detection, and fluorescence sensing. Initially, we modified the surface of the microwell array-based chips to improve the sampling process efficiency (Fig. 4d). The chips were treated using oxygen plasma and coated with three types of hydrophilic agents. This modification resulted in a significant increase in the sampling efficiency, especially when the chip was coated with P100 (> 99%) (Fig. 4e), and further experiments were conducted using P100. Additionally, we appropriately set the imaging module to determine the optimal working distance between the optical lens and the chip (Fig. 4f). By increasing the working distance, the image resolution decreased, whereas FoV increased. The detection rate remained high (> 99%) up to a 20-mm working distance, after which it gradually decreased. The 20-mm working distance was found to be the optimal distance at which the chip contour was clearly captured.

**Fig. 5:**
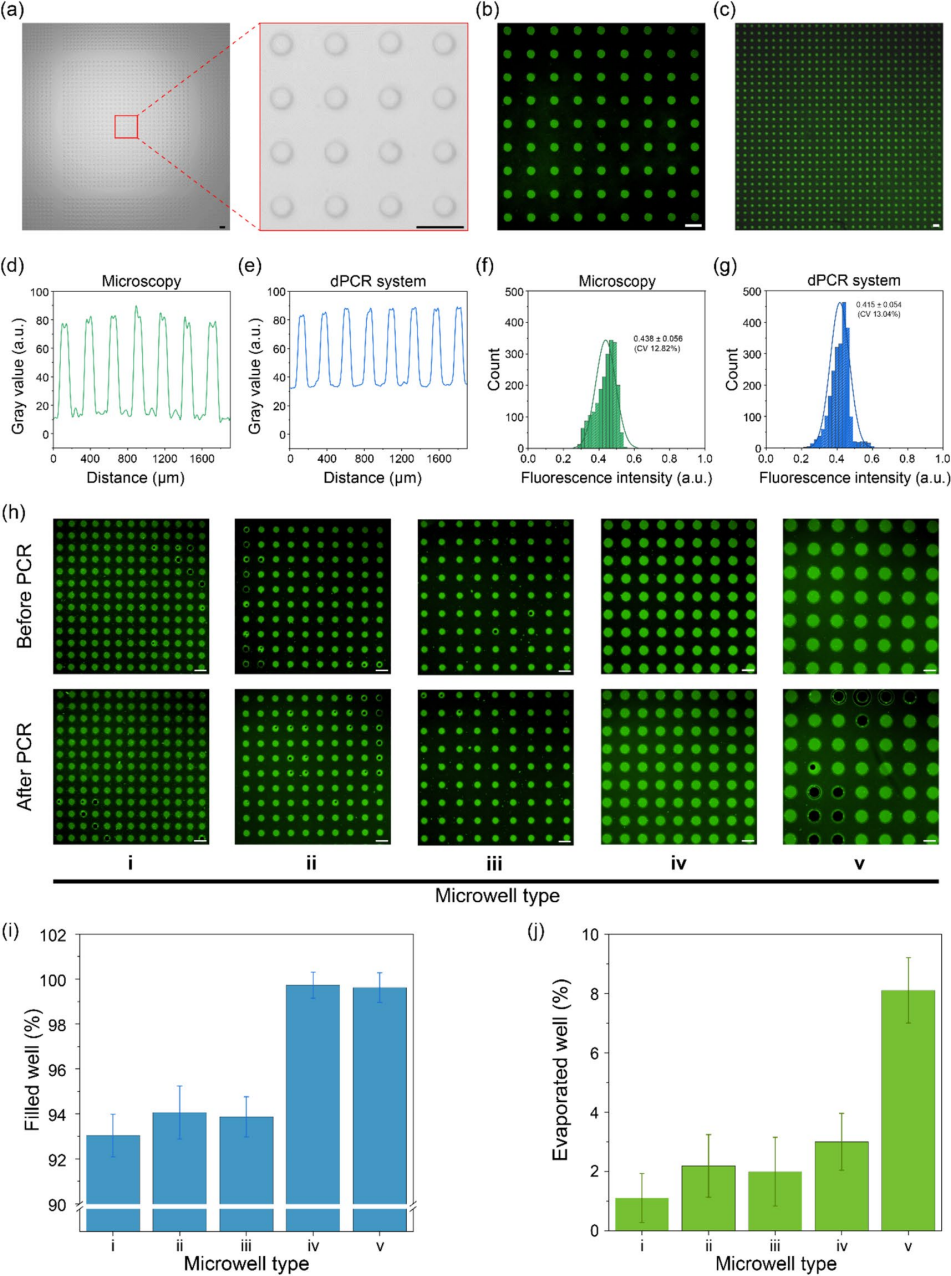
Characterization of the proposed dPCR system. (**a**) Bright-field images captured using the dPCR system. Fluorescence images captured using (**b**) a conventional bench-top microscope and (**c**) the dPCR system (scale bars: 200 μm). Line profiles demonstrating gray value fluctuations in images captured using (**d**) the microscope and (**e**) the dPCR system. Histograms showing the distribution of fluorescence intensities of the images captured using (**f**) the microscope and (**g**) the dPCR system; evaluation of the sample evaporation. (**h**) Fluorescence microscopy images of the fabricated microwell array-based chips before and after PCR thermal cycling for different microwell array types (i–v) (scale bars: 200 μm). Graphs representing the filled well ratio (i) before PCR thermal cycling and (j) after PCR thermal cycling

After determining the optimal working distance and capturing the corresponding bright-field images of the microwell array using the dPCR system (Fig. 5a), fluorescence images were captured using a conventional bench-top microscope and the dPCR system for an FITC-loaded chip to evaluate the fluorescence imaging performance (Fig. 5b-c). The fluorescence images were analyzed using line profiles to determine the observed fluctuations in gray values (Fig. 5d-e). The consistent variations in gray values indicated that the dPCR system can produce images with contrast and detail levels comparable with those of conventional systems. The measured fluorescence intensities were plotted on histograms (Fig. 5f-g). The dPCR system exhibited a uniform intensity distribution (CV = 13.04%), which is comparable with that of a conventional microscope (CV = 12.82%). Furthermore, it should be pointed out that the dPCR system offers a larger FoV (12.46 × 9.34 mm) than that of a fluorescence microscope (4.44 × 2.50 mm). Therefore, the optimized dPCR system can produce high-quality images with performance metrics comparable with those of conventional bench-top microscopes, verifying its effectiveness in accurate and reliable dPCR applications.

In dPCR assays, sample evaporation during thermal cycling is an important parameter for accurately determining the target DNA concentration. An FITC-diluted sample was placed on a fabricated microwell chip, and 40 PCR cycles were conducted (Fig. 5h). A low sampling efficiency was observed in microwells with relatively small diameters (types i–iii) (Fig. 5)). This can be attributed to the limited volume capacity and high surface tension that hinder efficient sample loading [43]. In contrast, the evaporation rate increased in microwells with relatively large diameters (type v) (Fig. 5j). This is probably due to large wells, which develop significant thermal gradients, resulting in high evaporation rates, even under sealed conditions during thermal cycling [44]. Consequently, we employed type iv microwells with optimal dimensions for the subsequent dPCR experiments. The final chip consisted of 25 panels, each comprising 30 × 30 microwells (22,500 in total) with a 150-µm diameter and a 300-µm pitch.

**Fig. 6:**
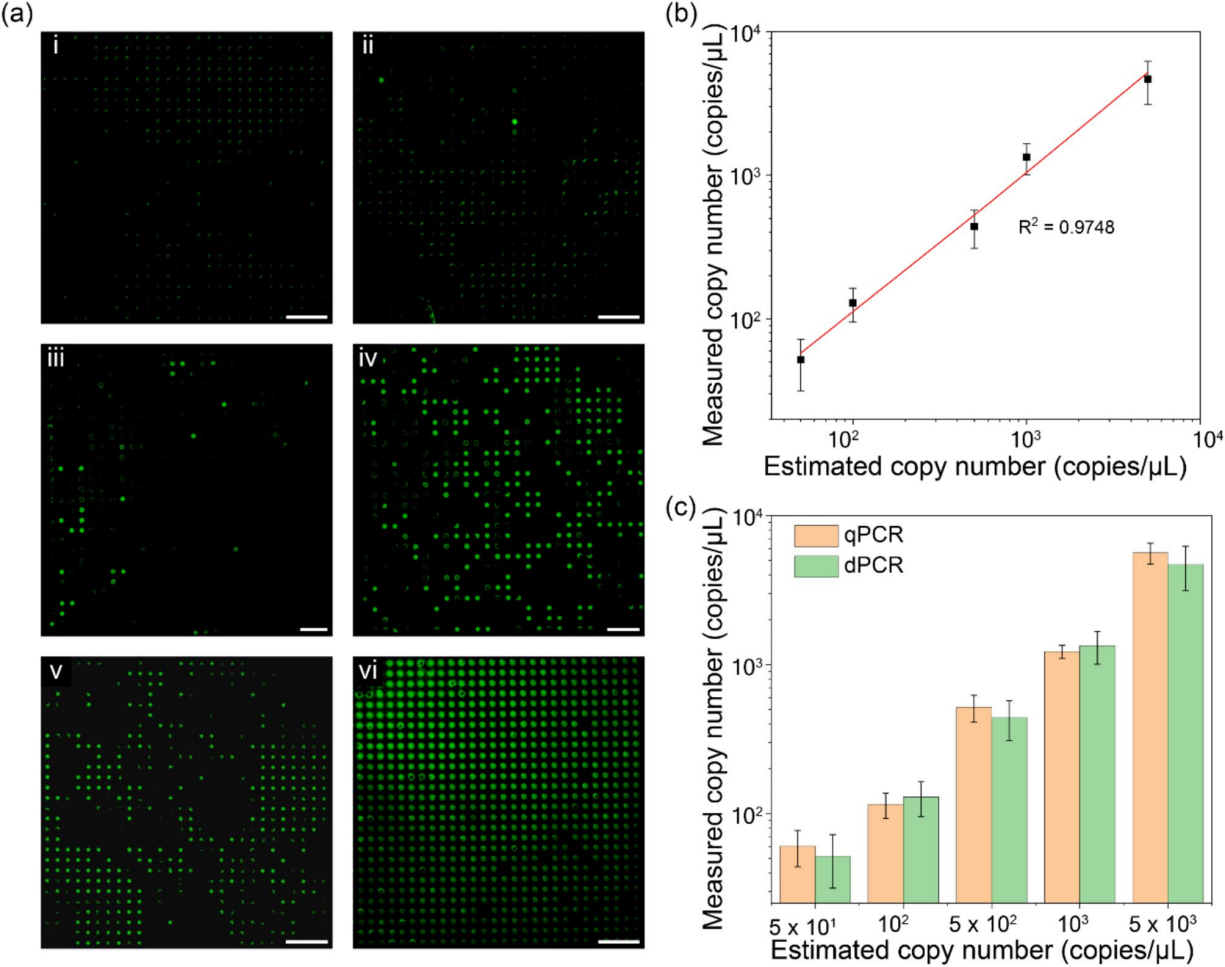
Determination of the human coronavirus 229E concentration. (**a**) Fluorescence microscope images demonstrating the dPCR assay results for (i) NTC and different DNA concentrations: (ii) 50, (iii) 100, (iv) 500, (v) 1,000, and (vi) 5,000 copies/µL. (**b**) Linearity graph showing the relationship between the estimated and calculated concentration of the target DNA. (**c**) Comparative analysis of quantification results between a commercial qPCR system and the proposed dPCR system (scale bars: 1 mm)

To evaluate the detection sensitivity and quantification accuracy of the developed dPCR system, we performed a series of experiments using cDNA samples of the human coronavirus 229E (HCoV-229E) with varying concentrations ranging from 50 to 5,000 copies/µL (Fig. 6). Figure 6a shows fluorescence images after DNA amplification for six different concentrations (0, 50, 100, 500, 1000, and 5,000 copies/µL). The ratio of positive wells was increased in proportion to the concentration of the target DNA, and no fluorescence signal was observed in the no-template control (NTC) sample. The concentration was predicted using the Poisson distribution based on the nominal volume of the microwells (0.88 nL) to verify the absolute quantification capability (Fig. 6b). In the 50–5,000 copies/µL concentration range, good agreement was observed between measured and estimated concentration (R^2^ = 0.9855), indicating the excellent detection accuracy of the system. To rigorously evaluate the accuracy and reliability of the dPCR system, we compared its performance with a commercially available qPCR system (Fig. 6c). We employed the same primers and probes and conducted thermal cycling steps under identical conditions to ensure consistency in all tests. The concentration measured using the developed dPCR system closely matched that of the qPCR system, demonstrating its reliability and accuracy in determining target molecule concentrations. Overall, the results demonstrated that the optimized microwell array-based chips and the developed integrated dPCR system can provide accurate, sensitive, and reliable quantification of the target DNA.

## Conclusions

In conclusion, we presented an advanced and reliable method for the mass production of microwell array-based dPCR chips and a portable battery-operated dPCR system. The proposed fabrication process, which is based on injection molding, enables the massive reproducibility and low cost of microwell array-based chips. Additionally, the photolithography-based metal mold fabrication process enables precise control over the size and transparency of microwells and is expected to expand the range of applications that require hole and pillar patterns at the micrometer scale. Future advancements could involve scaling down microwell dimensions to the nanometer range or exploring alternative shapes to enhance versatility and consider various applications, including high-throughput screening and single-cell analysis. The fabricated dPCR chips and the developed system exhibit high reproducibility and reliability in the analysis of infectious respiratory viruses. Using a portable and fluorescence signal analysis, the nucleic acid of a virus can be easily partitioned and placed inside individual microwells. After amplification, a strong green fluorescence signal can be easily observed and detected from 50 to 5,000 copies/µL. Overall, the proposed method provides an effective, reliable, sensitive, and cost-effective way to produce dPCR chips and systems for the analysis of a single copy level of nucleic acid. Moreover, the results are well-matched with those obtained using commercially available dPCR systems. We anticipate that our method is potentially applicable in POCT, thus improving public health.

## Data Availability

The datasets used and/or analyzed during the current study are available from the corresponding author on reasonable request.
